# Real-Life Assessment of the Ability of an Ultraviolet C Lamp (SanificaAria 200, Beghelli) to Inactivate Airborne Microorganisms in a Healthcare Environment

**DOI:** 10.3390/life13051221

**Published:** 2023-05-20

**Authors:** Claudio Foschi, Beatrice Giorgi, Simone Ambretti, Tiziana Lazzarotto, Francesco Saverio Violante

**Affiliations:** 1 Microbiology Unit, IRCCS Azienda Ospedaliero–Universitaria di Bologna, 40138 Bologna, Italy; claudio.foschi2@unibo.it (C.F.); simone.ambretti3@unibo.it (S.A.); 2 Section of Microbiology, Department of Medical and Surgical Sciences, University of Bologna, 40138 Bologna, Italy; 3 Division of Occupational Medicine, IRCCS Azienda Ospedaliero–Universitaria di Bologna, 40138 Bologna, Italy; beatrice.giorgi@aosp.bo.it (B.G.); francesco.violante@unibo.it (F.S.V.); 4 Occupational Medicine Unit, Department of Medical and Surgical Sciences, University of Bologna, 40138 Bologna, Italy

**Keywords:** UVC lamp, sanitizing device, airborne infections, germicidal effect, healthcare

## Abstract

Airborne-mediated microbial diseases represent one of the major challenges to public health. Ultraviolet C radiation (UVC) is among the different sanitation techniques useful to reduce the risk of infection in healthcare facilities. Previous studies about the germicidal activity of UVC were mainly performed in artificial settings or in vitro models. This study aimed to assess the sanitizing effectiveness of a UVC device (SanificaAria 200, Beghelli, Valsamoggia, Bologna, Italy) in ‘real-life’ conditions by evaluating its ability to reduce microbial loads in several hospital settings during routine daily activities. The efficacy of the UVC lamp in reducing the bacterial component was evaluated by microbial culture through the collection of air samples in different healthcare settings at different times (30 min–24 h) after turning on the device. To assess the anti-viral activity, air samplings were carried out in a room where a SARS-CoV-2-positive subject was present. The UVC device showed good antibacterial properties against a wide range of microbial species after 6 h of activity. It was effective against possible multi-drug resistant microorganisms (e.g., *Pseudomonas* spp., *Acinetobacter* spp.) and spore-forming bacteria (e.g., *Bacillus* spp.). In addition, the UVC lamp was able to inactivate SARS-CoV-2 in just one hour. Thanks to its effectiveness and safety, SanificaAria 200 could be useful to inactivate airborne pathogens and reduce health risks.

## 1. Introduction

Indoor air quality (IAQ) has a significant influence on the health, comfort, and well-being of building occupants [1]. One of the major sources of indoor air pollution is the presence of microorganisms, such as bacteria, viruses, and fungi, that are able to threaten people’s health in a more serious way than some organic and inorganic air contaminants [2].

In fact, airborne-mediated microbial diseases represent one of the major challenges to worldwide public health [3]. This aspect is particularly critical in healthcare settings/facilities for different reasons. First, several airborne bacterial pathogens, such as *Legionella pneumophila* and *Mycobacterium tuberculosis,* are known to cause severe illnesses and even fatalities in frail and/or immunocompromised hosts [2]. Second, under the selective pressure induced by the hospital environment, healthcare-associated infections are often due to multi-drug resistant microorganisms with high morbidity and mortality rates [4].

Moreover, viruses such as influenza and SARS-CoV-2, originally thought to be only transmitted from person to person via aerosols of body fluids, could remain suspended in the air and be spread to susceptible subjects through the production of infectious droplet nuclei [5,6].

Therefore, an effective and reliable disinfection system is required to inactivate airborne pathogens to maintain a good IAQ and reduce health risks.

In this context, several sanitation techniques have been developed and widely used to kill pathogenic microorganisms in the environment [7]. The airborne antimicrobial efficacy of ultraviolet (UV) light has long been established [8]. Germicidal UV light can also efficiently inactivate both drug-sensitive and multi-drug resistant bacteria, as well as differing strains of viruses [9,10]. The antimicrobial mechanism is mainly related to the absorption of UV by nucleic acid components: DNA/RNA damage and the formation of dimers between adjacent thymine in the DNA polynucleotide chains are the primary photoproducts of UV-exposed DNA [11].

Among the UV spectra, UVC radiation at 254 nm proved to effectively reduce the risk of infection in operating rooms of healthcare facilities, thanks to its germicidal activity [12,13]. However, UVC radiation at 254 nm can be dangerous for human health, potentially being the cause of eye (e.g., irritation and inflammation of the cornea) and skin (i.e., erythema) injuries [14].

Recently, the company Beghelli S.p.A. (Valsamoggia, Bologna, Italy) developed a UVC lamp (SanificaAria 200) with a new technology that eliminates the health risks due to UVC exposure. This germicidal UVC lamp can sanitize the environment in a few hours and can be safely used in the presence of people during daily activities, even in very crowded settings (i.e., hospitals, schools, and restaurants).

In this study, we assessed the sanitizing effectiveness of the UVC device SanificaAria 200 in ‘real-life’ conditions, evaluating its ability to reduce microbial loads (including bacteria and the SARS-CoV-2 virus) in hospital settings during routine daily activities.

Previous studies about the disinfection efficacy of UVC light were mainly carried out in artificial settings (e.g., a room-sized chamber) or in vitro models [15,16,17]. Thus, our approach is particularly intriguing since it mimics what happens in a ‘natural’ healthcare setting, where several factors (e.g., type and number of subjects, air exchange, ‘open systems’ with the outside) can affect the results.

## 2. Materials and Methods

### 2.1. Sanitation Device

The SanificaAria 200 (Beghelli; Valsamoggia, Bologna, Italy) is a UVC lamp that consists of a system of axial fan aspiration for air treatment by means of two ray lamps (cartridges) of ultraviolet in the “C band” (UVC).

The air present in the environment is aspirated and emitted inside a closed chamber in which the UVC source is active and where the sanitization process is carried out; subsequently, the sanitized air is expelled and returned to the environment. Detailed technical features of the sanitation device are displayed in the Supplementary material (Supplementary Table S1).

Preliminary experiments performed in artificial/controlled settings highlighted the excellent antimicrobial effectiveness of the device. In particular, when tested against *Serratia marcescens* (ATCC 13880) and *Bacillus subtilis* (ATCC 6633), SanificaAria 200 showed antibacterial activity of 99% and 90%, respectively. Moreover, the UVC device was able to significantly reduce the viral loads of Adenovirus-5 (−1.25 log) and of Coronavirus HCoV-OC43 (−2.5 log) (confidential data).

### 2.2. Air Samplers

Two different devices were used for air sampling: one for bacterial components and one more suitable for viral particles. In particular, for the sampling of bacteria, a Trio.Bas Duo cable contact plate air sampler was used (Orum International, Milan, Italy), equipped with two stainless steel aspirant heads (standard flow rate 200 L/min per head) and 65 mm contact plates of tryptic soy agar (kept at 2–8 °C before use).

A Coriolis Micro air sampler (Bertin Technologies SAS, Montigny-le-Bretonneux, France) (flow rate maximum 300 L/min for 10 min of sampling) equipped with sterile collection cones previously pre-filled with 7.5 mL of a saline phosphate buffer solution (PBS) was used to collect viral components.

### 2.3. Sampling Settings

The experiments were conducted between November 2021 and March 2022, and the study was conducted according to the regulations of the Ethical Committee of ‘IRCCS Azienda Ospedaliero-Universitaria’ of Bologna (Italy) and to the 1964 Helsinki Declaration and its later amendments.

To evaluate the efficacy of the UVC lamp in reducing the bacterial component, air sampling was performed in two different settings: the first in a hospital room of a private clinic in the Bologna urban area (Italy) (room volume = 40 m^3^) (Experiment A) and the second in a hospital room used as a library of the Unit of Occupational Medicine of IRCCS Azienda Ospedaliero-Universitaria of Bologna (room volume = 110 m^3^) (Experiment B). In both rooms where an active ventilation system was present, the windows were always closed, whereas the doors were opened/closed according to the needs of the personnel. The experiments were conducted during the routine daily activities of patients and medical/nursing staff. The air temperature was comprised between 20 and 22 °C, with a humidity rate of 25–30%.

Trio.Bas Duo sampler, equipped with two contact plates placed in the stainless-steel aspirant heads, was placed in the centre of the rooms on a table at eye level during sampling. The flow rate was 200 L/min, and the sampling time was 15 min. At the end of the sampling, contact agar plates were incubated at 37 °C for 48 h in an aerobic atmosphere.

To assess the anti-viral activity of the UVC sanitation system, air sampling was performed in an isolated room (windows and door closed), where a symptomatic subject with confirmed molecular positivity for the SARS-CoV-2 virus (COVID-19 disease) was present (room volume = 50 m^3^) (Experiment C).

The Coriolis Micro air sampler was placed in the centre of the room on a table at eye level; the flow rate was 300 L/min, and the sampling time was 10 min. Sterile cones were pre-filled with 7.5 mL of a solution of PBS and frozen at −20 °C after sampling before the analysis.

A preliminary evaluation was carried out (see Supplementary Table S2), repeating the sampling in the rooms at different time points throughout the day with UVC sanitizer switched off. In this way, we identified the time of day when the microbial loads were highest, which was established as the moment of the subsequent experiments with UVC lamps (see Supplementary Table S3).

Afterward, the SanificaAria 200 device was installed in the centre of the hospital room, switched on at medium speed, and maintained active until the end of the experiment (its effectiveness was tested over 24 h). At night, the sanitizer was set at a low speed to allow patients to sleep.

To evaluate the efficacy of reducing microbial levels over time, air samplings were carried out at different times after turning on the SanificaAria 200 device. The following time points were considered: before the activation of the lamp (t0) (starting time: 11:00 am), after 30 min of activity of UVC sanitizer (t1), after 1 h (t2), after 2 h (t3), after 4 h (t4), after 5/6 h (t5), after 22 h (t6), and after 24 h (t7). For each time point, for the bacterial component, the sampling time was 15 min.

### 2.4. Sample Analysis

First, we performed a quantitative evaluation of bacteria by counting colonies grown on contact agar plates. Colony count (expressed as colony-forming unit, CFU/m^3^) was carried out in duplicate using the Quantica 500 (Bioavlee, Wrocław, Poland) automatic colony counter and the Scan 100 (Interscience, Paris, France) manual colony counter. The number of colonies detected before the ignition of the SanificaAria 200 device was compared to the number of colonies found at different time points after the use of the UVC lamp. Results were expressed as the mean log CFU/m^3^ ± standard deviation (SD). A significant reduction in colony count was searched for using the one-way analysis of variance (ANOVA) test, followed by Dunnett’s Multiple Comparison test. Statistical significance was determined at *p* < 0.05 (*), *p* < 0.01 (**), and *p* < 0.0001 (***).

To achieve bacterial species-level identification, each colony with a different morphology was isolated on blood agar plates. After overnight incubation, colonies were identified using MALDI–TOF mass spectrometry (Bruker Daltonics, Bremen, Germany), as previously reported [18].

For the viral component, samples were analysed by a commercial multiplex nucleic acid amplification technique (BIOFIRE^®^ Respiratory 2.1 plus panel; BIOFIRE FILMARRAY Respiratory Panel; BioMerieux, Marcy-l’Étoile, France), following the manufacturer’s instructions. This PCR system detects the presence of 19 viruses that cause respiratory tract infections in 45 min and proved to be suitable/valid for SARS-CoV-2 virus detection [19]. The antiviral activity of the UVC device was evaluated as viral clearance ability (no molecular viral detection after the use of the UVC lamp).

## 3. Results

### 3.1. Activity of the UVC Device in Reducing Bacterial Levels

**Figure 1:**
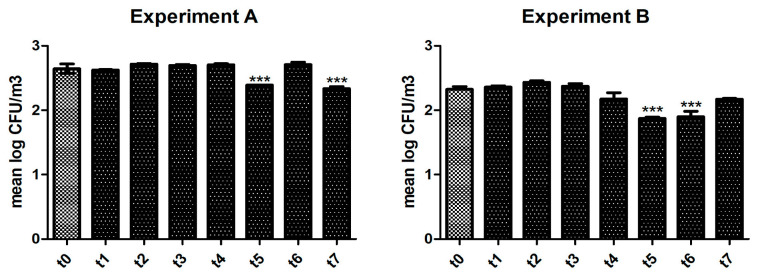
Antibacterial activity of the UVC lamp. The efficacy of reducing bacterial colonies was evaluated in two different healthcare settings, as described in the text: Experiment A (**left panel**) and Experiment B (**right panel**). Air samplings were performed at different time points after switching on the UVC device. The number of colonies (expressed as mean log CFU/m^3^) was compared to the control (i.e., absence of UVC activity). Statistical significance was searched for by the ANOVA test, followed by Dunnett’s Multiple Comparison test. *** *p* < 0.0001.

Figure 1 shows the result of Experiment A: a significant (*p* < 0.0001) reduction of bacterial loads was observed after 6 h (t5) and after 24 h (t7) of activity of the UVC device. As shown in Supplementary Table S4, between sampling t5 and t6, the sanitizer was set at minimum speed for the night period, thus potentially explaining the ‘rebound’ in colony count found. As displayed in Table 1, the air sampled before the activity of the UVC lamp was mainly rich in Gram-positive cocci or bacilli, such as staphylococci, micrococci, and *Bacillus* spp. The presence of some of these microorganisms (e.g., *Bacillus licheniformis* and *Bacillus cereus*) was completely abolished after 5 h of sanitizing activity and maintained over time.

During Experiment B (Figure 1), we observed a significant reduction (*p* < 0.0001) in colony count starting 5–6 h (t5) after the ignition of the UVC lamp. The efficacy was retained for the subsequent sampling (i.e., after 22 h, t6). Supplementary Table S5 shows the environmental conditions of the room during the air samplings. The list of bacteria identified before and after the use of the UVC device is displayed in Table 2. In addition to several species of Gram-positive bacteria (corinebacteria, staphylococci, micrococci, and *Bacillus* spp.), we were able to detect Gram-negative opportunistic rods. Some of these microbes completely disappeared 5–6 h after the ignition of the lamp (e.g., *Pseudomonas aeruginosa* and *Acinetobacter lwoffii*), whereas other bacterial species (e.g., *Klebsiella pneumoniae*) were detected only in the air sampled after the device activity.

### 3.2. Antiviral Activity of the UVC Device

The air sampling performed in a room with a COVID-19-positive subject showed molecular positivity for the SARS-CoV-2 virus (Experiment C; Supplementary Figure S1). Already one hour after switching on the UVC lamp, the virus was no longer detected by the molecular panel (Supplementary Figure S1). The negative result for the SARS-CoV-2 virus was retained for all the other subsequent samplings.

## 4. Discussion

In this study, we assessed the disinfection/antimicrobial activity of a UVC lamp during routine daily activities in a hospital setting.

At first, it is worth noting that air samples belonging to healthcare environments can be ‘contaminated’ by a plethora of different microbial species. In fact, we had the chance to identify both Gram-positive and Gram-negative cocci and rods, including staphylococci, *Bacillus* spp., Enterobacterales, and non-fermentative Gram-negative bacilli. Most of them can be opportunistic pathogens responsible for severe infections, such as respiratory tract and bloodstream infections, mainly in immunocompromised patients or in the presence of invasive medical devices [20,21]. Moreover, in healthcare settings, most of these bacteria show alarming patterns of antimicrobial resistance, with high morbidity and mortality rates and poor therapeutic options [22,23].

Thus, an effective, safe, and reliable sanitizing system is crucial to inactivate airborne pathogens and reduce health risks.

The UVC lamp SanificaAria 200 (Beghelli) proved to have good sanitizing ability against both bacteria and viruses. In fact, after only 6 h of activity, the device showed highly significant efficacy in reducing bacterial components based on the different microbial species. Interestingly, the UVC device was effective even against possible multi-drug resistant microorganisms (e.g., *Pseudomonas* spp., *Acinetobacter* spp.) and possible spore-forming bacteria (e.g., *Bacillus* spp.) [24,25]. In addition, regarding the viral component, a total abolishment of SARS-CoV-2 detection was obtained in just one hour.

This aspect is of particular importance if we consider the impact of the pandemic of SARS-CoV-2-associated coronavirus disease 2019 (COVID-19) worldwide, with approximately 15% of patients requiring hospitalization in intensive care units [26].

Previously published studies mainly investigated the antimicrobial activity of UVC lamps in in vitro models. Thus, it is not surprising that UVC lamps were found to be valid germicidal devices, showing excellent antimicrobial activities against a wide range of pathogens in a very short time [2,15,16,17,27].

A critical comparison of our results with previously published papers regarding the germicidal effect of UVC devices is not easy. Indeed, there is a high degree of variability in terms of experimental settings, protocols, and technologies among the different types of UVC sanitizing methods used to assess antimicrobial activity [28,29].

In our experiments, we noticed that the sanitation process was influenced by normal working conditions: an increase in bacterial colonies was recorded in some moments of the day, for example, in the presence of a continuous transit of people in and out of the room (i.e., after medical visits, during meals, etc.). Sometimes, this finding was combined with the appearance of bacterial species not identified in the previous air samplings. These results are not surprising if we consider that the composition of the air changes continuously in space and time based on several factors that are not easily controlled. Nevertheless, our goal was to demonstrate the efficacy of the UVC device in real-life conditions, mimicking what usually happens during hospital daily activities.

We are fully aware of some limitations of this study: (i) considering that the internal fan of the UVC device can create air movement, enhancing the air circulation and the resuspension of bioaerosols from the floor and other surfaces, it would have been necessary to monitor the air flow rate of the outlet of the device and estimate the possible effect of air disturbances; (ii) although the sampling time for the bacterial component was only 15 min for each time point, we cannot completely rule out a reduction of the viable bacteria concentration due to natural decay or destruction by oxidation, desiccation, or other phenomena.

Further additional studies are needed to better evaluate the cost-effectiveness of the use of SanificaAria 200 (Beghelli) in healthcare settings as well as to assess its efficacy in different experimental conditions (e.g., prolonged time points, activity against other microorganisms, additional experiments to evaluate the exact effectiveness against specific bacterial genera, other assays in artificial/controlled settings).

## 5. Conclusions

The UVC device SanificaAria 200 (Beghelli) showed good antibacterial properties against a wide range of microbial species after 6 h of activity in different healthcare real-life settings. In addition, the UVC lamp was able to inactivate SARS-CoV-2 in just one hour. Thanks to its effectiveness and safety, this device could be very useful to inactivate airborne pathogens and reduce health risks, making it a good option in settings where frail patients are hospitalized (e.g., intensive care units).

## Figures and Tables

**Table 1:** List of bacterial species identified during Experiment A. Identification at the species level was achieved by MALDI–TOF MS.

Bacteria Identified in Preliminary Sampling (No Activity on the Device)	Bacteria Identified after Switching on the Sanitizer for 5 h	Bacteria Identified after Switching on the Sanitizer for 24 h
*Bacillus cereus*	-	-
*Bacillus licheneformis*	-	-
*Micrococcus luteus*	*Micrococcus luteus*	*Micrococcus luteus*
*Staphylococcus capitis*	-	-
*Staphylococcus epidermidis*	*Staphylococcus epidermidis*	*Staphylococcus epidermidis*
*Staphylococcus haemolyticus*	*Staphylococcus haemolyticus*	*Staphylococcus haemolyticus*
*Staphylococcus hominis*	*Staphylococcus hominis*	*Staphylococcus hominis*

**Table 2:** List of bacterial species identified during Experiment B. Identification at the species level was achieved by MALDI–TOF MS.

Bacteria Identified in Preliminary Sampling (no Activity of the Device)	Bacteria Identified after Switching on the Sanitizer for 6 h
*Acinetobacter lwoffii*	*Acinetobacter lwoffii*
*Bacillus badius*	-
-	*Bacillus megaterium*
-	*Bacillus simplex*
*Corynebacterium afermentans*	-
-	*Klebsiella pneumoniae*
-	*Kocuria rizhophila*
*Micrococcus lylae*	*-*
*Micrococcus luteus*	*Micrococcus luteus*
*Moraxella osloensis*	*Moraxella osloensis*
*Pseudomonas aeruginosa*	-
*Staphylococcus epidermidis*	-
*Staphylococcus haemolyticus*	*Staphylococcus haemolyticus*
*Staphylococcus hominis*	*Staphylococcus hominis*

## Data Availability

All data underlying the research are presented in the text and in the Supplementary Material.

## Supplementary Materials

The following supporting information can be downloaded at: https://www.mdpi.com/article/10.3390/life13051221/s1, Table S1: Technical features of SanificaAria 200 (Beghelli); Table S2: Example of the work diagram related to preliminary samplings in a hospital room of a private clinic in the Bologna urban area. Table S3: Number of bacterial colonies detected during preliminary samplings; Table S4: Details about Experiment A. Table S5: Details about Experiment B. Figure S1: Activity of the UVC device against SARS-CoV-2.
