# Supplementary material for: Real-Life Assessment of the Ability of an Ultraviolet C Lamp (SanificaAria 200, Beghelli) to Inactivate Airborne Microorganisms in a Healthcare Environment
Source: Life (Basel). 2023 May 20;13(5):1221. doi: 10.3390/life13051221 (PMC10224295; doi:10.3390/life13051221)
Supplement: Supplementary file 1 [file life-13-01221-s001.zip › life-2372946-supplementary.pdf]

Supplementary materials

Supplementary Table S1. Technical features of SanificaAria 200 (Beghelli)

Technical features	Value
Power supply	90-253W, 50÷60Hz
Maximum absorption	<75W
UV-C Lamp	2 X TC 2G11 24W (total 48W)
Ozone free UV-C Lamp	/
UV-C Lamp radiant power	14W
UV-C wavelength	254 nm
Lamp life	12 months (depending on use)
Weight	13 kg (10 kg without bracket)
Device equipped with Bluetooth and WIFI System for remote control	/
Product classification	The product can be classified as an air purifier according to IEC 60335-2-65: 2002 "Household appliances and similar appliances - Safety - Part 2-65: particular requirements for air purification equipment in compliance with (paragraph 32.102) "

Fan speed	Flow of treated air	Noise level
I	81 m^3/h	38dB(A) to 1 meter
II	130 m^3/h	47dB(A) to 1 meter
III	200 m^3/h	59dB(A) to 1 meter

Supplementary Table S2. Example of the work diagram related to preliminary samplings in a hospital room of a private clinic in Bologna urban area

Monday 6/12/21 from 9.05 to 9.20<br>Room number 211<br>Two patients present<br>Room measurements 3x4,5x3 m (40 m^3)	***Monday 6/12/21 from 11.10 to 11.25***<br>Room number 211<br>One patient and two nurses present	***Monday 6/12/21 from 12.55 to 13.10***<br>Room number 211<br>Two patients present	***Monday 6/12/21 from 16.00 to 16.15***<br>Room number 211<br>Two patients present
Tuesday 7/12/21 from 9.20 to 9.35<br>Room number 213<br>Two patients present<br>Room measurements 3x4,5x3 m (40 m^3)	***Tuesday 7/12/21 from 11.20 to 11.35***<br>Room number 213<br>One patient presents	***Tuesday 7/12/21 from 13.10 to 13.25***<br>Room number 213<br>Two patients, one physiotherapist and one nurse present	***Martedì 7/12/21 from 15.45 to 16.00***<br>Camera number 213<br>Two patient present
Thursday 9/12/21 from 9.20 to 9.35<br>Room number 206<br>Two patients present<br>Room measurements 3x4,5x3 m (40 m^3)	***Thursday 9/12/21 from 11.10 to 11.25***<br>Room number 206<br>One patient presents	***Thursday 9/12/21 from 12.55 to 13.10***<br>Room number 206<br>One patient presents	***Thursday 9/12/21 from 15.55 to 16.10***<br>Room number 206<br>One patient presents
Friday 10/12/21 from 9.20 to 9.35<br>Room number 224<br>One patient presents	***Friday 10/12/21 from 11.15 to 11.30***<br>Room number 224<br>Two patients present	***Friday 10/12/21 from 13.05 to 13.20***<br>Room number 224<br>Two patients present	***Friday 10/12/21 from 15.50 to 16.05***<br>Room number 224<br>Two patients present

Room measurements 4,5x4,5x3 m (60 m^3)			
Monday 13/12/21 from 9.50 to 10.05<br>Room number 219<br>Two patients not present<br>Room measurements 4,5x4,5x3 m (60 m^3)	***Monday 13/12/21 from 11.30 to 11.45***<br>Room number 219<br>Two patients	***Monday 13/12/21 from 13.10 to 13.25***<br>room number 219<br>Two patients present	***Monday 13/12/21 from 15.55 to 16.10***<br>Room number 219<br>Two patients present

Supplementary Table S3. Number of bacterial colonies detected during preliminary samplings.

The moment of the day when the bacterial load was the highest is highlighted in yellow.

	Number of colonies (expressed as CFU/m^3)			
**Date**	9:00 AM	11:15 AM	01:00 PM	04:00 PM
6/12/21	76	215	105	95
7/12/21	46	86	70	70
9/12/21	130	125	72	81
10/12/21	117	173	190	159
13/12/21	36	89	64	48

Supplementary Table S4. Details about Experiment A.

Sampling	People present**	Speed SanificaAria 200***
t_0	2 patients + physiotherapist	Lamp off
t_1	2 patients (lunch time)	Medium
t_2	2 patients (lunch time)	Medium
t_3	2 patients	Medium
t_4	1 patient	Medium
t_5	2 patients	Medium
t_6	2 patients	Medium
t_7	1 patient	Medium

At the end of the preliminary sampling, the sanitizer was switched on at medium speed.

** Between samples 5-6 and 7-8 the door was always closed. For samples 1-2-3-4 the door was kept open several times by the staff for service reasons.

*** Between sampling 6 and 7, the sanitizer was set at minimum speed, to reduce background noise, favouring rest.

Supplementary Table S5. Details about Experiment B.

Sampling	People present	Speed SanificaAria 200
t0	6 people	Lamp off
t1	4 people	Medium
t2	7 people	Medium
t3	2 people	Medium
t4	1 person	Medium
t5	2 people	Medium
t6	2 people	Medium
t7	1 person	Medium

Supplementary Figure S1. Activity of the UVC device against SARS-CoV-2. The upper panel shows the detection of SARS-CoV-2 before the ignition of the device. Already one hour after switching on the UVC lamp, the virus was no longer detected (lower panel)

BioFire®
Respiratory Panel 2.1 *plus*

BIO FIRE
BY BIOMÉRIEUX
www.BioFireDx.com

Run Summary

**Sample ID:**	ARIA 1	**Run Date:**	17 Mar 2022<br>2:54 PM
**Detected:**	Severe Acute Respiratory Syndrome Coronavirus 2 (SARS-CoV-2)	**Controls:**	Passed
**Equivocal:**	None		

Result Summary

	**Viruses**
Not Detected	Adenovirus
Not Detected	Coronavirus 229E
Not Detected	Coronavirus HKU1
Not Detected	Coronavirus NL63
Not Detected	Coronavirus OC43
Not Detected	Middle East Respiratory Syndrome Coronavirus (MERS-CoV)
✓ Detected	Severe Acute Respiratory Syndrome Coronavirus 2 (SARS-CoV-2)
Not Detected	Human Metapneumovirus
Not Detected	Human Rhinovirus/Enterovirus
Not Detected	Influenza A
Not Detected	Influenza B
Not Detected	Parainfluenza Virus 1
Not Detected	Parainfluenza Virus 2
Not Detected	Parainfluenza Virus 3
Not Detected	Parainfluenza Virus 4
Not Detected	Respiratory Syncytial Virus
	**Bacteria**
Not Detected	*Bordetella parapertussis* (IS*1001*)
Not Detected	*Bordetella pertussis* (*ptxP*)
Not Detected	*Chlamydia pneumoniae*
Not Detected	*Mycoplasma pneumoniae*

Run Details

**Pouch:**	RP2.1*plus* v1.0	**Protocol:**	NPS2 v3.2
**Run Status:**	Completed	**Operator:**	Laboratorio Virologia (virologia)
**Serial No.:**	54817128	**Instrument:**	TM09140
**Lot No.:**	29G021		

BioFire®
Respiratory Panel 2.1 *plus*

BIO FIRE
A BIOMÉRIEUX COMPANY
www.BioFireDx.com

Run Summary

**Sample ID:**	ARIA 2	**Run Date:**	17 Mar 2022<br>2:39 PM
**Detected:**	None	**Controls:**	Passed
**Equivocal:**	None		

Result Summary

	**Viruses**
Not Detected	Adenovirus
Not Detected	Coronavirus 229E
Not Detected	Coronavirus HKU1
Not Detected	Coronavirus NL63
Not Detected	Coronavirus OC43
Not Detected	Middle East Respiratory Syndrome Coronavirus (MERS-CoV)
Not Detected	Severe Acute Respiratory Syndrome Coronavirus 2 (SARS-CoV-2)
Not Detected	Human Metapneumovirus
Not Detected	Human Rhinovirus/Enterovirus
Not Detected	Influenza A
Not Detected	Influenza B
Not Detected	Parainfluenza Virus 1
Not Detected	Parainfluenza Virus 2
Not Detected	Parainfluenza Virus 3
Not Detected	Parainfluenza Virus 4
Not Detected	Respiratory Syncytial Virus
	**Bacteria**
Not Detected	*Bordetella parapertussis* (IS*1001*)
Not Detected	*Bordetella pertussis* (*ptxP*)
Not Detected	*Chlamydia pneumoniae*
Not Detected	*Mycoplasma pneumoniae*

Run Details

**Pouch:**	RP2.1*plus* v1.0	**Protocol:**	NPS2 v3.2
**Run Status:**	Completed	**Operator:**	virologia virologia (virologia)
**Serial No.:**	54817132	**Instrument:**	2FA07396
**Lot No.:**	29G021		
